# A decline in the energy content of forage fish in the Bay of Biscay

**DOI:** 10.1111/jfb.70116

**Published:** 2025-06-18

**Authors:** Morgane Amelot, Marianne Robert, Maud Mouchet, Dorothée Kopp

**Affiliations:** ^1^ Centre d'Ecologie et des Sciences de la Conservation, UMR 7204 MNHN‐CNRS‐Sorbonne Université Paris France; ^2^ DECOD L'Institut Agro, IFREMER, INRAE Lorient France

**Keywords:** Bay of Biscay, energy, feeding behaviour, lipids, prey

## Abstract

Planktonic production fuels the base of marine food webs and can mediate energy accumulation in consumers but is affected by several anthropogenic pressures. Our understanding of how shifts in prey quality at various trophic level shape marine food webs remains poor. This study explores variation in the nutritional quality of prey of hake *Merluccius merluccius* and whiting *Merlangius merlangus* between the 2000s and 2020s. An overall decrease in prey quality was observed between these periods. Prey species previously considered high quality no longer differed in nutritional composition from intermediate‐quality prey species. These results suggest that a profound trophic reorganization is currently taking place in the Bay of Biscay, probably mediated through bottom‐up processes.

Global change impacts marine ecosystems through different processes, including climate change, pollution and fishing pressure (Koenigstein et al., 2016). Because these pressures are exerted on different trophic levels, whether directly (e.g., fishing mortality) or indirectly (e.g., species displacements caused by global warming), they could result in ecosystem reorganization (Pörtner & Peck, 2010). For instance, from the mid‐1980s, fertilizer legislation in Europe led to a decrease in nutrient inputs to the North‐East Atlantic, resulting in variation in phytoplankton production (Desmit et al., 2020). In addition, global warming has been associated with variation in zooplankton and phytoplankton quality (Hixson & Arts, 2016).

Such shifts in resource quality have had important consequences for the condition of small pelagic fish since the beginning of the 2000s, particularly in North‐East Atlantic food webs (Doray et al., 2018; Véron et al., 2020). These species, such as sardines *Sardina pilchardus* (Walbaum 1792), anchovy *Engraulis encrasicolis* (L.) or mackerel *Scomber scombrus* (L.), feed on planktonic organisms. As a result, their recent decline in length and body condition was hypothesized to be related to changes in zooplankton and phytoplankton production (Marquis et al., 2006).

Although consequences of variation in planktonic production have been studied extensively for low trophic level species such as small pelagic fish (Peck et al., 2021), there is less information on responses of higher trophic‐level species to prey quality deterioration.

Among high trophic‐level species, Gadiformes (Corrales et al., 2022; Day et al., 2019) have seen large changes in diet in recent years (Amelot et al., 2023a; Amelot et al., 2023b). More specifically, hake *Merluccius merluccius* (L.) and whiting *Merlangius merlangus* (L.) have switched from being selective piscivores with a dominance of high‐quality fish prey such as small pelagics in the 1980s (Belleggia et al., 2019; Mahe et al., 2007; Pinnegar et al., 2003; Shaw et al., 2008) to being generalist omnivores, including a mix of fish and invertebrate prey, in the 2010s. Selective species consume certain prey more than others in accordance with energy trade‐offs, which are determined by prey abundance, swimming and hiding capacities and nutritional quality (Scharf et al., 2000; Spitz et al., 2018), whereas opportunist species consume prey based on their local abundance. Because fish have generally higher swimming capacities than invertebrates, a decrease in their nutritional quality as prey could have modified the energy trade‐offs that previously motivated selective predatory behaviour towards them (Meyers et al., 2021). This could have caused a change in *M. merluccius* and *M. merlangus* diets through a shift from selectively preying on fish to opportunist feeding behaviour.

Our objectives in this study were (1) to determine whether *M. merluccius* and *M. merlangus* prey nutritional quality has changed over the past 20 years; and (2) to see whether any such changes impacted particular prey quality groups and could therefore support the hypothesis of a shift in the diets of *M. merluccius* and *M. merlangus*.

The Bay of Biscay is located from 1 to 10° west and from 43 to 48° north, opening onto the North‐East Atlantic Ocean. Diet reports from a stomach content study (Pinnegar, 2014) and metabarcoding databases (Lejeune et al., 2022) were used to define the potential prey of *M. merluccius* and *M. merlangus* in the area (Appendix: Table S1). From these potential prey, a set of 16 species was selected to estimate prey nutritional quality evolution.

Prey species were sampled in the Bay of Biscay during the EVHOE (*EValuation des ressources Halieutiques de l'Ouest de l'Europe*) and ORHAGO (*Observation of the benthic aquatic resources of the GOlfe de Gascogne*) campaigns (Coupeau, 2023; Laffargue et al., 2022; Laffargue et al., 2023) in October and November 2022 and 2023. Data collected on these samples were then compared to those published in Spitz et al. (2010) based on sampling by the same campaigns conducted in October and November. The data from Spitz et al. (2010) were collected between 2002 and 2008, but the number of year covered by species varied from 1 to 2 years as it did for the contemporary period. Samples were collected with a demersal trawl and kept frozen until they could be processed in the laboratory. Efforts were made to sample comparable size classes between periods, but the opportunistic nature of the sampling resulted in size range discrepancies for some species. A subset of data was selected to reduce this inter‐period size variability and test if it would have impacted the general results (Appendix: Table S2); no change in energy content trends was observed.

To reduce interindividual variability, contemporary samples were created for analysis by pooling the entire bodies of several individuals of each species (Table 1). These pools of specimens were then ground into a homogenous material from which 40 g was sent to the Labocea laboratory for further analyses. As some past samples had only one replicate per species, it was not possible to assess variability between replicates.

**TABLE 1:** Mean nutritional content of the species in the different prey quality groups by period, 2002–2008 energy results are from Spitz et al. (2010).

Quality groups based on Spitz et al. (2010)	Species	Species code	Period	Length (cm)	Individuals sampled (number of replicates)	Water % [min–max]	Protein % [min–max]	Lipids % [min–max]	Ash % [min–max]	Energy kJ.g^−1^ [min–max]
High (HQ)	*Scomber scombrus*	SCOM	2002–2008	25–29	12 (4)	67.3 [66.4–69.9]	17.5 [17.3–17.8]	10.5 [7.9–13.6]	2.1 [1.8–2.4]	7.9 [7.1–8.5]
			2022–2023	18–27	15 (5)	71.8 [67.2–76.0]	19.3 [18.8–19.7]	6.3 [2.4–10.9]	10.1 [2.3–3.4]	5.6 [4.2–7.3]
	*Trachurus trachurus*	TRAC	2002–2008	14–30	30 (5)	72.4 [71.1–74.0]	18.2 [17.3–19]	5.0 [3.6–6.2]	3.1 [2.4–4.5]	6.0 [5.6–6.5]
			2022–2023	15–19	15 (5)	77.0 [76.3–78.9]	17.5 [16.6–17.9]	2.0 [1.2–2.7]	3.7 [3.5–3.8]	3.8 [3.3–4.0]
	*Sardina pilchardus*	SARD	2002–2008	12–22	15 (3)	65.3 [63.2–67.4]	17.8 [16.7–19.1]	11.7 [8.4–17.1]	2.4 [1.8–3.3]	8.7 [7.5–10.1]
			2022–2023	16–17	15 (5)	76.9 [76.5–77.6]	18.6 [17.9–19.0]	1.6 [0.7–2.4]	4.1 [3.8–4.5]	3.8 [3.5–4.1]
	*Argentina sphyraena*	ARGE	2002–2008	11–16	22 (2)	72.2 [71.5–72.2]	17.8 [16.5–19.0]	5.7 [5.2–6.1]	2.5 [2.5–2.5]	6.1 [6.0–6.2]
			2022–2023	11–19	18 (5)	73.8 [71.0–76.8]	17.2 [16.4–17.8]	6.7 [4.1–8.9]	3.4 [3.1–3.7]	5.4 [4.4–6.3]
	*Atherina presbyter*	ATHE	2002–2008	5–12	129 (3)	67.8 [65.9–68.8]	19.8 [18.9–21.2]	7.3 [6.6–8.1]	3.0 [2.1–3.7]	7.3 [7.1–7.5]
			2022–2023	7–11	46 (5)	75.2 [73.1–76.8]	18.1 [17.6–18.6]	3.0 [2.2–4.2]	4.1 [4.0–4.5]	4.2 [3.9–4.7]
	*Capros aper*	CAPR	2002–2008	6–7	36 (1)	71.3	17.2	4.8	4.6	6.2
			2022–2023	6–10	58 (4)	74.1 [72.3–74.9]	16.6 [15.8–17.2]	4.3 [3.1–5.5]	6.0 [5.4–6.3]	4.4 [4.0–4.9]
	*Engraulis encrasicolus*	ENGR	2002–2008	9–13	208 (4)	72.0 [69.0–76.0]	19.6 [18.2–20.3]	3.4 [1.7–5.2]	2.8 [1.9–3.2]	5.8 [4.9–6.7]
			2022–2023	12–14	25 (5)	75.4 [73.8–76.5]	19.3 [19.1–19.6]	1.9 [1.1–3.4]	3.1 [2.9–3.2]	4.1 [3.7–4.6]
	*Sprattus sprattus*	SPRA	2002–2008	7–13	246 (4)	70.9 [69.1–75.4]	17.2 [16.4–18.2]	8.2 [3.4–11.2]	2.5 [2.3–2.7]	6.5 [4.8–7.3]
			2022–2023	8–12	24 (5)	68.1 [67.0–69.0]	16.5 [16.0–16.8]	14.0 [11.3–14.1]	2.7 [2.6–2.9]	7.6 [7.0–8.1]
Intermediate (IQ)	*Merlangius merlangus*	MERL	2002–2008	17–22	24 (4)	79.5 [79.3–79.7]	16.7 [16.6–16.9]	0.7 [0.3–1.0]	2.9 [2.3–3.5]	3.9 [3.8–3.9]
			2022–2023	14–23	15 (5)	79.1 [77.7–80.3]	17.8 [16.6–18.6]	1.3 [0.6–2.5]	3.0 [2.6–3.3]	3.5 [3.2–4.0]
	*Trisopterus minutus*	TRIS	2002–2008	14–18	21 (3)	73.8 [71.5–75.3]	18.8 [17.9–20.3]	2.8 [2.4–3.3]	3.5 [2.3–2.4]	5.1 [5.0–5.2]
			2022–2023	14–18	15 (5)	73.6 [72.1–74.8]	19.1 [18.3–20.1]	2.6 [1.9–3.2]	4.3 [3.2–5.5]	4.4 [4.2–4.7]
	*Micromesistius poutassou*	MICR	2002–2008	14–20	40 (4)	77.9 [77.0–78.3]	17.4 [16.1–18.1]	1.5 [1.1–1.7]	3.1 [2.2–4.0]	4.4 [4.0–4.7]
			2022–2023	17–20	14 (5)	75.7 [74.0–76.1]	18.4 [17.9–19.3]	1.9 [1.2–2.4]	3.8 [3.3–4.9]	3.9 [3.5–4.2]
	*Callionymus lyra*	CALL	2002–2008	15–19	5 (1)	75.4	17.1	2.0	3.6	5.2
			2022–2023	10–21	17 (5)	76.8 [75.8–78.3]	17.4 [16.5–18.4]	1.8 [0.6–2.9]	4.1 [3.6–4.4]	3.7 [3.2–4.1]
	*Lesueurigobius friesii*	LEUS	2002–2008	4–6	143 (1)	72.4	16.5	4.1	4.8	5.6
			2022–2023	4–8	90 (3)	74.9 [74.6–75.1]	16.5 [16.2–16.9]	4.6 [4.4–4.7]	4.2 [4.0–4.3]	4.5 [4.5–4.5]
	*Pasiphaea sivado*	PASI	2002–2008	4–9	342 (1)	78.1	17.6	0.5	3.4	4.1
			2022–2023	2–9	130 (4)	79.0 [78.0–79.8]	16.3 [15.6–16.7]	1.2 [1.0–1.3]	3.0 [2.9–3.1]	3.3 [3.2–3.4]
Low [LQ]	*Merluccius merluccius*	MELU	2002–2008	22–29	9 (3)	80.4 [79.5–81.2]	16.0 [15.1–16.9]	0.7 [0.3–1.1]	2.7 [2.2–3.2]	3.7
			2022–2023	26–29	9 (3)	79.7 [79.3–80.1]	17.0 [16.0–17.6]	1.0 [0.9–1.2]	3.4 [2.9–4.2]	3.27
	*Sepia officinalis*	SEPI	2002–2008	6–10	10 (2)	75.8 [75.7–75.9]	15.8 [15.3–16.4]	1.2 [1.0–1.4]	5.5 [5.2–5.8]	3.8
			2022–2023	4–9	15 (5)	78.0 [76.8–78.8]	16.0 [14.4–17.1]	0.9 [0.8–1.0]	4.4 [2.8–6.4]	3.21

*Note*: The ‘length’ column gives the length range (minimum–maximum) in centimetres. ‘Individuals sampled’ is the total number of individuals of a species sampled in a period. ‘Number of replicates’ is the number of pools that were treated separately for nutritional content estimation.

Proximate composition (water, ash, lipid, protein, carbohydrate contents) and energy content were determined to evaluate the nutritional quality of prey species. For water content, a part (5 g) of each of the biological samples was mixed with Fontainebleau sand and ethanol (95%), oven‐dried at 103 ± 2°C for a night and weighed (Afnor, 2001a). Ash content was determined by heating 5 g of the biological sample in a furnace for 4 h at 550 ± 25°C. This process eliminates organic materials by incineration, and the inorganic matter that remains is then weighed (Afnor, 2001b). For lipid determination, samples (2.5 g) were boiled in 3 N hydrochloric acid (100 mL) for 30–45 min. Then, the mixture was filtered through a filter composed of quartz sand and celite, and the filters dried in a microwave oven. Total lipid content was extracted by a solid–liquid extraction method (EU, 2009), using a Buchi B811Soxhlet extraction system with a petroleum‐ether solvent. The extracted lipids were collected in beakers and weighed. Total protein was inferred from the nitrogen content, following the Kjeldahl method (Afnor, 2002), whereby a portion (0.5 g of biological sample) was digested with sulfuric acid in the presence of a mineralization catalyst, and the liberated nitrogen retained as ammonium sulphate. Ammonia was then released from the acid digest by the addition of sodium hydroxide. The ammonia was distilled, collected in a boric acid solution and titrated with standardized hydrochloric acid N/10. Protein content was then estimated by multiplying nitrogen content by a conversion factor of 6.25. Carbohydrate content was calculated as follows: total carbohydrate content (%) = 100 – water content (%) – ash content (%) – lipid content (%) – protein content (%). Finally, energy was calculated (EU, 2011) based on the following equation: E (kJ/100 g) = [(17 × protein content (%) + (37 × lipid content (%) + (17 × total carbohydrate content (%)].

To characterize prey nutritional composition distribution between the periods 2002–2008 and 2022–2023, a correspondence analysis (CA) was performed on the standardized mean lipid, protein, ash, water and energy content by species and period, using quotient transformation via the *data. Normalization* function of *clusterSim* R package (Dudek & Walesiak, 2020).

According to Spitz et al. (2010), three prey quality groups were determined based on species’ past energy density: high quality (HQ, energy density >6 kJ g^−1^): *S. scombrus*, horse mackerel *Trachurus trachurus* (L.), *S. pilchardus*, argentine *Argentina sphyraena* L., sand smelt *Atherina presbyter* Cuvier 1829, boarfish *Capros aper* (L.), *Engraulis encrasicolus*, sprat *Sprattus sprattus* (L.); intermediate quality (IQ, 4 < energy density <6 kJ g^−1^): *M. merlangus*, poor cod *Trisopterus minutus* (L.), blue whiting *Micromesistius poutassou* (Risso 1827), common dragonet *Callionymus lyra* L., Fries's goby *Lesueurigobius friesii* (Malm 1874), white glass shrimp *Pasiphaea sivado* (Risso 1816); and low quality (LQ, energy density <4 kJ g^−1^): *M. merluccius*, common cuttlefish *Sepia officinalis* L. The co‐ordinates of the species by period were extracted from the first two axes of the CA to estimate a 75% ellipse by quality group using the *SIBER* R package (Jackson et al., 2011).

To estimate the separation of quality groups from one another, we used the *maxLikOverlap* function from the *SIBER* package (Jackson et al., 2011) to quantify the realized overlap between HQ and IQ groups, as the number of species in the LQ group was not enough to estimate an ellipse surface. Finally, we tested the probability of an increasing overlap, understood as a reduced isolation, between these two groups using the Bayesian ellipse overlap function *bayesianOverlap* from the *SIBER* package, with three chains, and 1000 iterations as burn‐in (number of initial iterations discarded). The increases in simulated 75% overlaps were statistically compared using a Student *t*‐test.

Large differences were observed between the studied periods for both lipids and energy content. In all species except *S. sprattus*, energy contents decreased over time (Table 1). This decrease in energy content was, on average, 33% for HQ group (excluding *S. sprattus*), 17% for IQ group and 14% for LQ group (Table 1). The lipid percentage also decreased between periods for all HQ species apart from *S. sprattus* and *A. sphyraena* (Table 1).

Based on their nutritional composition, the distribution of species on the CA shows a segregation based on the quality and the period (Figure 1a). When considering nutritional composition measured in 2002–2008, HQ species (on the left), apart from *E. encrasicolus* (ENGR), are separated from LQ and IQ species (on the right) along the *x*‐axis. Conversely, HQ, LQ and IQ species with nutritional composition measured between 2022 and 2023 remained clustered on the right side of the *x*‐axis, except for *S. sprattus* (SPRA) and *A. sphyraena* (ARGE).

**FIGURE 1:**
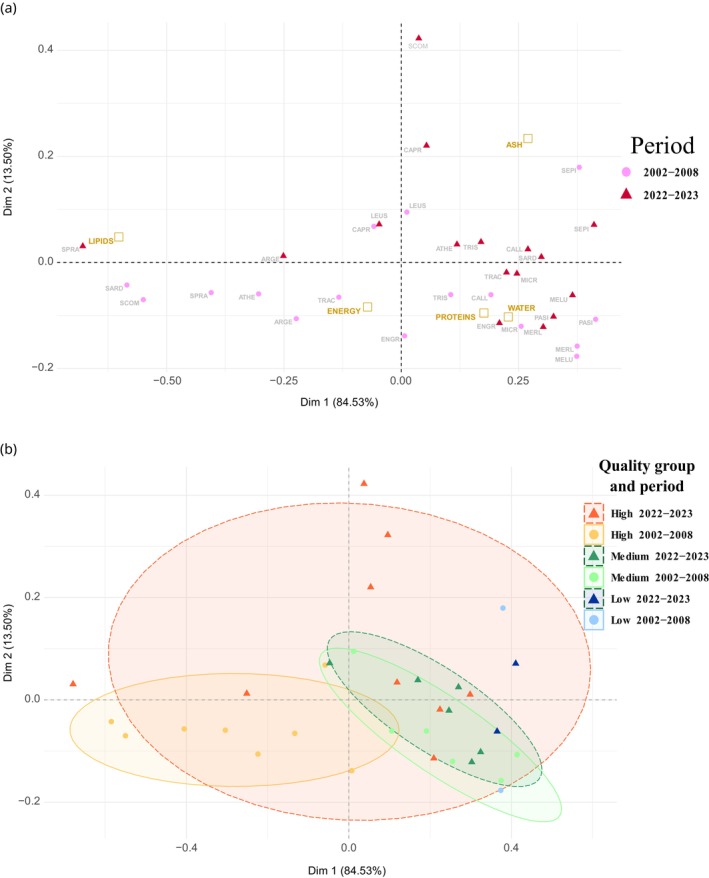
(a) Correspondence analysis outputs. Species (abbreviations given in Table 1) are represented by pink circles for the 2002–2008 period and red triangles for the 2022–2023 period. Nutritional components are represented by brown squares. (b) 75% ellipses are represented by prey quality group and period based on the correspondence analysis co‐ordinates.

The global and relative percentage of overlap between HQ and IQ group 75% ellipses increased significantly (*t*‐test, *p*‐value <2.2e−16) between periods. During the first period, the overlaps of the ellipse surfaces between groups were almost non‐existent: 4% and 8% for the HQ and IQ groups, respectively. During the second period, however, the overlap increased: 13%, for the HQ group and 98% for the IQ, including almost all of the ellipse surface (Figure 1b).

From the period of 2002–2008 to 2022–2023, the discrepancy between HQ and IQ/LQ groups fell drastically, associated to a decrease in energy content of HQ and IQ prey. These results expand previous findings on small pelagic fish energy content decrease (Doray et al., 2018; Véron et al., 2020) to broader forage species categories, inducing variations in energy gains for predators. However, information that could extend the period studied to better examine inter‐annual variability in trophic interactions would be highly valuable in understanding these variations in energy transfer.

Energy gain from prey consumption is the result of a balance between the energy expended to consume a prey and the energy content of this prey that can be assimilated by the predator (Giacomini, 2022; Sutton & Arnould, 2022). As such, all else being equal (e.g., diet composition, prey–predator size ratio, prey density and distribution), a reduction in prey energy contents would have a direct impact on predator energy gain (McCluskey et al., 2016; von Biela et al., 2019).

A first option is that *M. merluccius* and *M. merlangus* could have maintained the same diets despite the decrease in prey quality content, leading to a reduction in energy gain. Alternatively, these predators could have changed their diet, a phenomenon observed in these same species in the Celtic Sea during the same period (Amelot et al., 2023a). None of the prey species of *M. merluccius* and *M. merlangus* increased in energy content during this period apart from *S. sprattus*. Consequently, neither of these options would be beneficial to *M. merluccius* or *M. merlangus*, although the second could potentially limit their energy losses through a decrease in hunting costs. Indeed, the HQ group includes a majority of small pelagic fish known to show predator avoidance behaviour that could incur high energy costs for predators chasing them (Lambert et al., 2019).

Fish diet represents the entry point of most of the basic elements underlying individual body condition and functioning (Currie & Evans, 2020), with different roles in fish metabolic functions. Lipids, constituted of fatty acids, contribute to locomotion, growth and reproductive functions (Tocher, 2003), whereas protein contributes mainly to growth and reproductive functions (Craig et al., 2017). The energy gained through prey consumption is a proxy of these different elements. Variations in prey composition are likely to result in differential energy allocation by predators (Albo‐Puigserver et al., 2017; Persson & De Roos, 2006). In the earlier of the two periods studied, separation of the HQ group from the other groups was mainly related to high lipid content in these species. Lipids mainly enter marine food webs through phytoplankton production (Jónasdóttir, 2019; Napiórkowska‐Krzebietke, 2017). As such, a decrease in prey lipid composition is likely to be related to bottom‐up processes. Variations in phytoplankton production in relation to climate change have been explored through variation in phytoplankton abundance, species assemblages and proxy composition (Edwards et al., 2022; Galloway & Winder, 2015; Hixson & Arts, 2016). As stated in the introduction, total phytoplankton production did not decrease over our study period (Desmit et al., 2020). Phytoplankton lipid quantity is mainly controlled by the species that constitute the community (Galloway & Winder, 2015). These assemblages have been found to vary in coastal environments in relation to temperature (Carstensen et al., 2015; Derolez et al., 2020). Finally, lipid production variation within species has been tested in controlled experiments and demonstrates a decrease in lipid production when phytoplankton are exposed to warmer temperatures (Titocci & Fink, 2024). It has also been suggested that zooplankton production has been impacted by climate change due to variation in zooplankton abundance, lipids and energy content (Garcia et al., 2024; Titocci & Fink, 2024). These changes would have had an impact on the quality of the zooplankton consumed by planktivorous fish such as small pelagics (Bachiller & Irigoien, 2015; Chouvelon et al., 2015).

This study highlighted important shifts in hake and whiting prey nutritional quality over the past 20 years, mainly characterized by a decrease in quality of prey defined as highly qualitative in the past. Both the mechanistic understanding of the factor that could cause those changes in prey quality and the trade‐off processes that could result from such change would require complementary information. On one side, details of phytoplankton production in offshore areas or trophic transfer would provide support to understand potential bottom‐up processes at stage in the Bay of Biscay. On the other side, additional information on predator–prey relationship through detailed handling time and diet variations along periods could inform possible energetic trade‐off driving the Bay of Biscay trophic structure.

## AUTHOR CONTRIBUTIONS

M. A.: funding acquisition, conceptualization, methodology, data analysis and writing, M. R.: conceptualization, supervision, validation, M. M.: conceptualization, supervision, validation, D. K.: funding acquisition, conceptualization, methodology, supervision, validation.

## FUNDING INFORMATION

This study was part of the M&M project supported by France Filière Pêche (24/1004144).

## Supporting information


**Data S1:** Supporting information.
