# Supplementary material for: A decline in the energy content of forage fish in the Bay of Biscay
Source: J Fish Biol. 2025 Jun 18;107(4):1447–53. doi: 10.1111/jfb.70116 (PMC12536050; doi:10.1111/jfb.70116)

# Appendix : A decline in the energy content of forage fish in the Bay of Biscay

#### **Authors**

Morgane Amelot ^1,2^, Marianne Robert ^2^, Maud Mouchet ^1^, Dorothée Kopp ^2^

**Corresponding author**: morgane.amelot@mnhn.fr

#### **Affiliations**

^1^ Centre d'Ecologie et des Sciences de la Conservation, UMR 7204 MNHN-CNRS-Sorbonne Université, Paris, France

^2^  DECOD, L'Institut Agro, IFREMER, INRAE, 56100, Lorient, France

**Table 1** Diet of hake (*Merluccius merluccius*) and whiting (*Merlangius merlangus*) in the Bay of Biscay based on the Stomach content database (Pinnegar, 2014) and barcoding data (Lejeune et al., 2022). Minimum number of prey individuals were considered for the stomach content database, while this information is missing for barcoding data. Only species also sampled in Spitz et al., 2010 were sampled for the contemporary period.

| Predator | Year | Prey | Minimum number of prey | Zone | Method |
| --- | --- | --- | --- | --- | --- |
| *Merlangius merlangus* | 1991 | *Argentina sphyraena* | 2 | VIIa | Binocular |
|  |  | *Sardina pilchardus* | 1 | VIIa | Binocular |
|  |  | *Trachurus trachurus* | 1 | VIIa | Binocular |
| *Merluccius merluccius* | 1983 | *Portunus* spp. | 1 | VIIa | Binocular |
|  |  | *Sardina pilchardus* | 1 | VIIa | Binocular |
|  |  | *Argentina sphyraena* | 2 | VIIa | Binocular |
|  |  | *Micromesistius poutassou* | 15 | VIIa | Binocular |
|  |  | *Trisopterus minutus* | 1 | VIIa | Binocular |
|  |  | *Trachurus trachurus* | 2 | VIIa | Binocular |
|  | 1984 | *Micromesistius poutassou* | 14 | VIIa | Binocular |
|  |  | *Trisopterus minutus* | 1 | VIIa | Binocular |
|  | 1986 | *Argentina sphyraena* | 1 | VIIa | Binocular |
|  |  | *Cyclothone braueri* | 1 | VIIa | Binocular |
|  |  | *Micromesistius poutassou* | 123 | VIIa | Binocular |
|  |  | *Trisopterus esmarki* | 1 | VIIa | Binocular |
|  |  | Macrouridae | 1 | VIIa | Binocular |
|  |  | *Trachurus trachurus* | 5 | VIIa | Binocular |
|  | 1988 | *Sardina pilchardus* | 1 | VIIa | Binocular |
|  |  | *Micromesistius poutassou* | 5 | VIIa | Binocular |
|  |  | *Capros aper* | 1 | VIIa | Binocular |
|  | 1991 | Amphipoda | 5 | VIIa | Binocular |
|  |  | *Micromesistius poutassou* | 3 | VIIa | Binocular |
|  |  | Brachyura-Cancridea | 1 | VIIa | Binocular |
|  |  | Crangonidae | 2 | VIIa | Binocular |
|  |  | Crustacea | 3 | VIIa | Binocular |
|  |  | *Sepia officinalis* | 1 | VIIa | Binocular |
|  |  | Euphausiidae | 18 | VIIa | Binocular |
|  |  | *Argentina sphyraena* | 9 | VIIa | Binocular |
|  |  | *Scomber scombrus* | 2 | VIIa | Binocular |
|  |  | *Sardina pilchardus* | 14 | VIIa | Binocular |
|  |  | *Pandalus* spp*.* | 1 | VIIa | Binocular |
|  |  | Caridea | 6 | VIIa | Binocular |
|  |  | *Alpheus glaber* | 1 | VIIa | Binocular |
|  |  | *Trisopterus minutus* | 4 | VIIa | Binocular |
|  | 1992 | *Trachurus trachurus* | 1 | VIIa | Binocular |
|  | 1993 | *Merluccius merluccius* | 1 | VIIa | Binocular |
|  |  | Myctophidae | 1 | VIIa | Binocular |
|  |  | *Pandalus* spp. | 1 | VIIa | Binocular |
|  |  | *Trachurus trachurus* | 1 | VIIa | Binocular |
| *Merlangius merlangus* | 2019 | *Atherina* spp. | 1 | VIIa | Barcoding |
|  |  | *Trachurus trachurus* | 1 | VIIa | Barcoding |
|  |  | Clupeidae | 1 | VIIa | Barcoding |
|  |  | Blenniidae | 1 | VIIa | Barcoding |
|  |  | *Eugraulis encrasicolus* | 1 | VIIa | Barcoding |
|  |  | *Merluccius merluccius* | 1 | VIIa | Barcoding |
|  |  | *Pleuronectes platessa* | 1 | VIIa | Barcoding |
|  |  | Gobidae | 1 | VIIa | Barcoding |
|  |  | *Sardina pilchardus* | 1 | VIIa | Barcoding |
|  |  | *Sprattus sprattus* | 1 | VIIa | Barcoding |
|  |  | *Trisopterus* spp. | 1 | VIIa | Barcoding |
|  |  | *Alosa* spp. | 1 | VIIa | Barcoding |
|  |  | *Mullus surmuletus* | 1 | VIIa | Barcoding |
|  |  | *Crangon* spp. | 1 | VIIa | Barcoding |
|  |  | *Palaemon* spp. | 1 | VIIa | Barcoding |
|  |  | *Necora puber* | 1 | VIIa | Barcoding |
|  |  | *Gastrosaccus spinifer* | 1 | VIIa | Barcoding |
| *Merluccius merluccius* | 2019 | *Atherine* spp*.* | 1 | VIIa | Barcoding |
|  |  | *Clupeidae* | 1 | VIIa | Barcoding |
|  |  | *Engraulis encrasicolus* | 1 | VIIa | Barcoding |
|  |  | Gadidae | 1 | VIIa | Barcoding |
|  |  | *Sardina pilchardus* | 1 | VIIa | Barcoding |
|  |  | *Solea solea* | 1 | VIIa | Barcoding |
|  |  | *Sprattus sprattus* | 1 | VIIa | Barcoding |
|  |  | *Alosa* spp. | 1 | VIIa | Barcoding |
|  |  | *Callionymus* spp. | 1 | VIIa | Barcoding |
|  |  | *Pollachius* spp. | 1 | VIIa | Barcoding |
|  |  | *Trisopterus* spp*.* | 1 | VIIa | Barcoding |
|  |  | *Atelecyclus* spp. | 1 | VIIa | Barcoding |
|  |  | *Crangon* spp. | 1 | VIIa | Barcoding |
|  |  | *Necora* spp. | 1 | VIIa | Barcoding |
|  |  | *Gastrosaccus* spp. | 1 | VIIa | Barcoding |

**Table 2** Prey energy content by species for the whole set of data as well as a subset selection, while removing out of size range data (pooled date did not offered the opportunity to completely fit size classes). The difference between all data and subset data were calculated, as well as the difference between period for the (1) all data and (2) subset data alone.


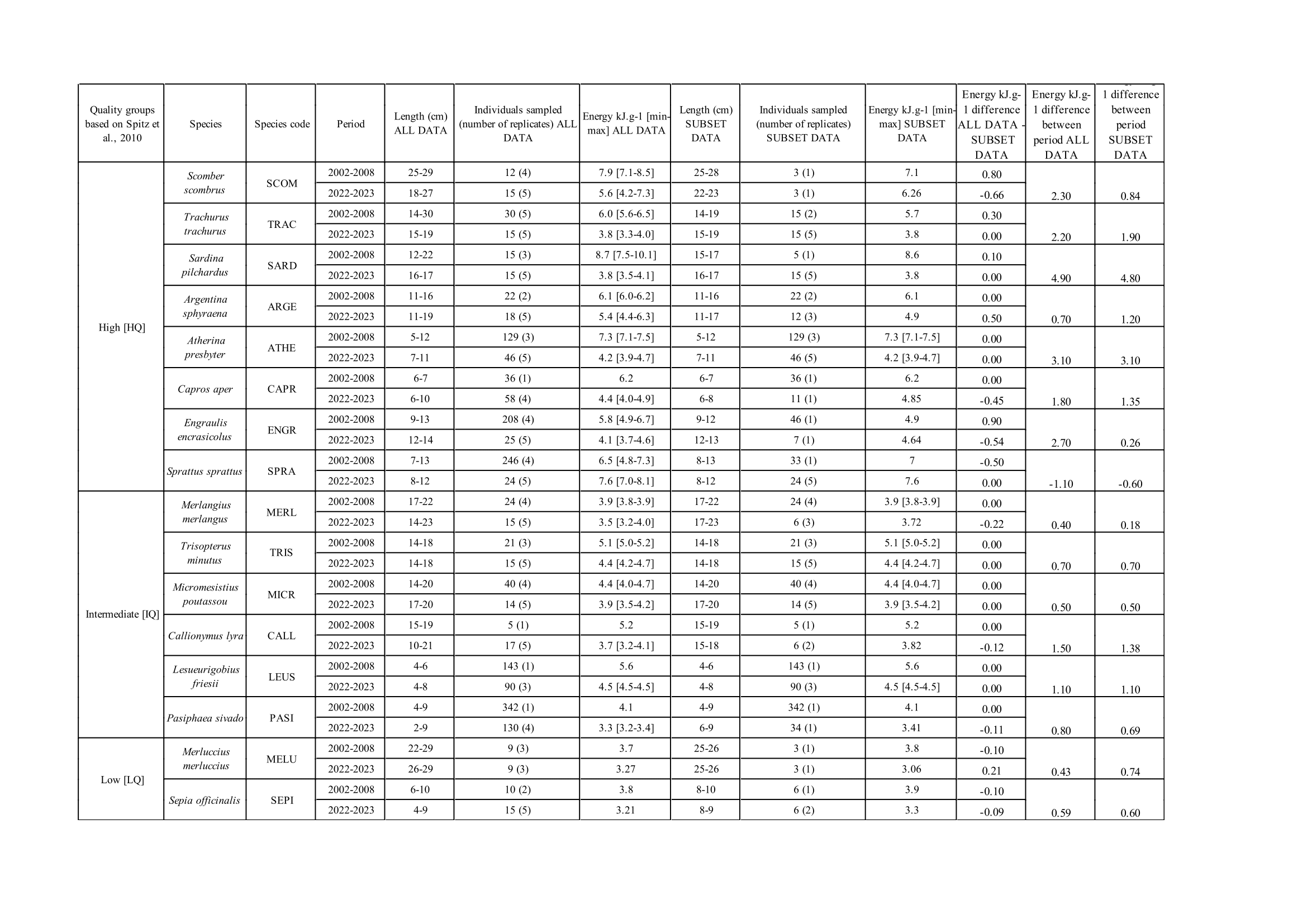

Supplement: Supplementary file 1 — Data S1: Supporting information. [file JFB-107-1447-s001.docx]
